# SWOT analysis of a physical activity intervention delivered to outpatient adults with a mild traumatic brain injury

**DOI:** 10.1177/20503121231166638

**Published:** 2023-04-17

**Authors:** Christophe Alarie, Isabelle Gagnon, Lily Trang Thao Huynh, Karine Doucet, Adèle Pichette-Auray, Cassandre Hinse-Joly, Bonnie Swaine

**Affiliations:** 1 École de Réadaptation, Faculté de Médecine, Université de Montréal Montréal, QC, Canada; 2 Centre for Interdisciplinary Research in Rehabilitation of Greater Montreal (CRIR), Montréal, QC, Canada; 3 Institut Universitaire sur la Réadaptation en Déficience Physique de Montréal (IURDPM), Montréal, QC, Canada; 4 School of Physical and Occupational Therapy, Faculty of Medicine, McGill University, Montréal, QC, Canada; 5 Trauma Center and Pediatric Emergency Medicine, Montreal Children’s Hospital, McGill University Health Center and Research Institute of the McGill University Health Center, Montréal, QC, Canada

**Keywords:** Physical activity, exercise, mild traumatic brain injury, concussion, rehabilitation, SWOT analysis

## Abstract

**Objectives:**

Physical activity interventions are effective to reduce the symptoms and recovery time after a mild traumatic brain injury; such interventions are not always embedded in all interdisciplinary outpatient settings. Service providers of a specialized rehabilitation program recognized the need to implement emerging evidence-based approaches to improve physical activity delivery. Understanding the perceptions of managers, clinicians, and users regarding the strengths, weaknesses, opportunities, and threats of the current physical activity intervention delivered to outpatient adults with a mild traumatic brain injury could inform local and widespread intervention development, enhancement, and implementation of evidence-based physical activity interventions.

**Methods:**

This study used a descriptive qualitative design using a strength, weakness, opportunity, and threat analysis framework. Managerial staff (*n* = 3), clinicians (*n* = 6), and program users (*n* = 5) with persisting symptoms following a mild traumatic brain injury from an outpatient specialized public rehabilitation program in Québec (Canada) participated. Individual semi-structured interviews were performed, recorded, transcribed verbatim, and analyzed using a qualitative content analysis approach.

**Results:**

Participants were generally positive about the intervention but expressed that improvement was required. Strengths (*n* = 15), weaknesses (*n* = 17), opportunities (*n* = 12), and threats (*n* = 6) related to eight overarching categories: physical activity intervention, health-related outcomes, clinical expertise, knowledge translation, communication, user engagement, resources, and accessibility. Category descriptions, convergent and divergent perspectives, and salient quotes of participants are provided.

**Conclusions:**

Participants were generally positive about the intervention (e.g., format) but identified weaknesses (e.g., need for service providers to better describe the physical activity intervention using theoretically driven approaches). Consultations of stakeholders will inform future intervention enhancement efforts and assist in ensuring interventions meet user needs.

## Introduction

Mild traumatic brain injury (mTBI) in adults may lead to persisting symptoms and become a disabling injury for months and even years.^1^ Slow-to-recover individuals of a mTBI may have reduced body functions (e.g., cognitive limitation, exercise intolerance, emotional, or sleep–wake disturbances), activity limitations (e.g., ability to walk or run), participation restrictions (e.g., work absenteeism, difficulty assuming social roles), and a reduced quality of life.^2–6^ Interdisciplinary management of mTBI may help reduce persisting symptoms and foster participation.^7,8^

Physical activity (PA) interventions have been shown to be an effective therapeutic approach to improve symptoms and reduce recovery time post mTBI.^9,10^ Some PA interventions are based solely on progressive submaximal aerobic exercise, while others are more complex and include multiple modalities such as oculovestibular, coordination, or visualization exercise in addition to aerobic exercise.^11–14^ Although the optimal exercise parameters (e.g., length of intervention, frequency, intensity, duration, type of exercise, and progression) to guide exercise prescription remain unknown,^14,15^ PA interventions are increasingly being recommended as part of the management of adults with persisting symptoms of mTBI.^7,8,16^

Specialized rehabilitation programs provide services to individuals with a mTBI on an inpatient and outpatient basis within the public healthcare system of Québec, Canada. Rehabilitation programs are interdisciplinary, and typically, inpatient programs are offered only to persons with complex mTBI (i.e., positive CT scan or polytrauma)^17^ and focus on improving body functions while outpatient programs aim to improve social reintegration. Although we do not know whether inpatient programs address persisting symptoms, a recent study with 16 clinical TBI programs within the province treating individuals with mTBI on an outpatient basis reported that only a third of them offer PA interventions to reduce persisting symptoms of a mTBI.^18^ Service providers of one particular TBI program with which our team of researchers has been collaborating was interested in reorganizing/restructuring their services before their program relocated across town. The service providers involved in the outpatient TBI program wished to evaluate the quality of certain aspects of their services, and in particular their PA intervention considering the growing evidence about the effectiveness of PA to reduce persistent mTBI symptoms and facilitate recovery following a mTBI.^9–10^

Knowledge about the organizational context in which a change would occur is key to inform planning and executing change.^19^ Evidence about PA interventions designed for adults with a mTBI in a hospital setting are limited^11,20–22^ and no study reports on effective PA intervention embedded in an interdisciplinary *outpatient* setting. Also, there was no approach or intervention in the literature that the team felt the service providers could adapt and integrate into their program that would meet the specific needs of their adult clientele with a mTBI. To inform quality improvement efforts intended for the near future, and changes about the inner workings of the intervention (e.g., specific exercise prescription parameters, format of delivery, goals of the intervention), the team of researchers and service providers felt it was necessary to learn about what worked and what did not work, regarding their current PA intervention.

Therefore, the aim of this study was to document stakeholders’ perceptions (i.e., social validity) about the quality of an existing PA intervention and of the changes needed to improve it. Results of this study will provide insights into the inner workings of an interdisciplinary outpatient PA intervention. Combined with the most up-to-date evidence about PA for adults with a mTBI, the current results may provide important information to stakeholders interested in creating a PA intervention or improving existing programs.

## Material and methods

The study employed a descriptive qualitative design to investigate the PA intervention currently provided to outpatient program users (i.e., adults with persisting symptoms of mTBI). This design was chosen to generate a comprehensive summary of the PA intervention in its natural state as perceived and experienced by individuals involved in the PA intervention without any attempt to manipulate or interfere with service delivery.^23,24^ The research question underpinning this study was as follows: *What are the perceptions of managers, clinicians, and users about their outpatient rehabilitation PA intervention designed for individuals with a mTBI?*

### Participants and sampling

Participants were service providers and program users recruited using purposive sampling to ensure information-rich cases.^25^ To be included in the study, the service providers (*n* = 9) needed to be involved directly or indirectly in the delivery of the PA intervention at the time of the study. Delivery of the PA intervention involved a program manager, two clinical coordinators, and six clinicians (e.g., occupational therapist, social worker, physiotherapist, kinesiologist). The program manager agreed to provide a short list of users who completed the PA intervention in the last 2 years prior to the study hoping not to overwhelm too many users with requests to participate in research. To be included in the study, program users must have received the PA intervention provided over the 2 years prior to the study by a physiotherapist, a kinesiologist, or the intern supervised by the kinesiologist. Exclusion criteria were not being able to do an in-person interview and not being sufficiently proficient in speaking either French or English. A research coordinator, internal to the institution, initially contacted potential participants by phone to determine their interest in participating, and then, the first author contacted the interested users by phone to schedule an interview. Only one list of 12 adult users with a mTBI was provided by the program manager. They were all contacted and five agreed to participate, a rate of participation typical for this rehabilitation center. The reasons for declining were not documented. All the participants were aware of the aims of the study and that it was part of the first author’s doctoral research.

### Setting

The TBI interdisciplinary rehabilitation program team provides specialized services annually to more than 100 adults with TBI including ≈70 individuals with mTBI living in Montréal, Québec and the surrounding area. To be eligible for the rehabilitation program, users with mTBI must present symptoms that were significantly impacting their participation for at least 3 months. They often have symptoms persisting for more than a year after the injury. The PA intervention embedded within the mTBI program is provided to persons with persisting symptoms of a mTBI in an individual manner or in small groups primarily by physiotherapists and kinesiologists. The PA intervention aimed to engage users with a mTBI in supervised low-to-moderate intensity PA by beginning with proprioception, balance, and mobility exercises. They could also walk outdoors and perform low intensity water-based PA (since the facility had pool access) while progressing to more complex exercises requiring bigger muscle groups and integrating visuo-ocular components, when needed. The prescription of the type of PA selected, and the frequency and the number of exercises are not explicitly described in a document, but exercise and PA progression are individualized to each program user with a mTBI based on the therapist’s judgment and clinical experience. Therapists report providing feedback and encouragement to promote pleasure and self-efficacy related to PA.

### Procedure

Data collection was performed from the Fall 2017 through to the Spring 2018, and analysis, interpreting, and reporting were conducted until 2022. To gather an important quantity of information and explore everyone’s perspective in depth, we used semi-structured interviews.^26^ Interview guides were developed around the SWOT (**s**trength, **w**eakness, **o**pportunity, and **t**hreat) analysis framework since it can help inform in planning small and large reorganization of health services.^26,27^ Perceptions about the strengths and weaknesses represent internal factors and attributes of the organization. Alternatively, the opportunities and threats represent external factors and attributes of the environment.^28^ Perceptions regarding the latter could represent common threats to rehabilitation programs across the province, given the programs are part of a network of care and funded by the provincial automobile insurance board. Two interview guides were developed for the study, one for service providers and a second one, slightly modified for users to facilitate their ability to talk freely about their own experience with the program. Guides contained one core question for each SWOT category, and associated sub-questions were used for further probing. For example, “In your opinion, what are the strengths of the current physical activity intervention provided (that you received from) to the program’s mTBI clientele? (Probe about strengths regarding the type of training, frequency of supervised sessions with clinicians, motivation, etc.)” (see Supplemental File I). To better tailor the interview guides toward service providers’ needs and to ensure consistency between the two versions, both guides were verified/enhanced for clarity by the program manager and pilot tested with two clinicians external to the TBI program.

Interviews were conducted by the first author (CA), a male doctoral student, and four female undergraduate students (CHJ, KD, APA, and LTTH) supervised by a senior scientist (BS), all external to the outpatient rehabilitation program. The first author is a kinesiologist with 5 years of clinical experience in private practice and the physiotherapy undergraduate students each had less than a year of clinical experience. The first author had an interest in using PA as a means to enhance the management of adults with a mTBI and aimed to develop and explore the feasibility and effectiveness of a new PA intervention during his doctoral studies. The first author hoped that the results of this study would inform the future development of a new PA intervention for outpatients with a mTBI. Interviewers were instructed to ask the four core questions but had flexibility to probe and to navigate the interviews to ensure a naturally flowing conversation. All interviewers used reflexivity through group discussions with the senior scientist (BS) to help them increase their awareness of their own subjectivity (e.g., values, opinions, experiences).^29^

Before performing the semi-structured interviews, the first author familiarized himself with the clinical environment of the TBI program by performing several on-site observations, attending interdisciplinary meetings, and shadowing a kinesiologist while delivering aspects of the PA intervention (since there was little to no written details about the intervention). Assessing program users’ health-related outcomes related to the PA intervention through direct measurements with standardized assessment tools was beyond the scope of this study. Observations were written as field notes and kept in a journal. Written informed consent and basic demographic information were obtained from participants (e.g., self-reported gender, age, years of clinical experience, mechanism of injury, time since injury). Interviews were conducted in person in French or English at the rehabilitation center or in a private and comfortable environment for some users (i.e., university office). There were no nonparticipants present during the interviews and no participants were interviewed twice. Interviews lasted less than an hour, were audio recorded, and transcribed verbatim. The transcripts were not returned to the participants for comment. Field notes were taken during and after the interviews and were kept in a journal. To ensure the quality of how interviews were conducted and of participants’ responses, the senior scientist (BS) listened to the recorded interviews of the first two service providers and of the first program user. This enabled her to provide feedback to the interviewers about how to improve the fluidity and probing depth. Minor adjustments were made to the interview guides (e.g., more probes).

### Statistical analysis/data analysis

Data from all interviews were analyzed using a semi-deductive qualitative content analysis approach using Microsoft Word software.^30^ The analysts (CA, CHJ, KD, APA, and LTTH) familiarized themselves with the data by conducting multiple readings of the interview transcripts and discussed preliminary thoughts during group discussions. Relevant passages in the transcripts of both service providers and program users with a mTBI were coded using a four-level coding notation based on SWOT categories. For example, if a user reported having trouble communicating with a service provider, the code *Weakness_Communication* was attributed. Each transcript was coded independently, and a second coder verified each transcript coding for correctness. The second coder infrequently suggested coding changes. When changes were suggested, they were minor and resolved through group discussion under the supervision of the senior author. When all verbatim were coded, related codes were aggregated and organized in mutually exclusive categories. Using this approach, 50 categories were generated resulting in 15 strengths, 17 weaknesses, 12 opportunities, and 6 threats. Further inspection of these categories revealed that perceptions of service providers and program users could be further grouped into eight broader categories (see Section “Results”). The combination of the recruitment challenges, the specificity of the sample, and the quality of the dialogue with the adult users with a mTBI allowed for a smaller sample without reducing the “power” of the information gathered.^31^ Also, given the small sample size and our desire to preserve anonymity, we did not specify who provided the quote when reporting citations.^32^

To further enhance trustworthiness, debriefing sessions with coauthors and senior researchers (BS and IG) were held throughout the analysis to improve interpretations and content of the codes, categories, and overarching categories.^33^ The participants did not provide feedback on the findings. An audit trail was built over the duration of the study including interview guide development information, process notes about methodology and trustworthiness, reflexive notes, raw data, and products of analysis and synthesis.

## Results

One program manager, two clinical coordinators, six clinicians and five users were interviewed (*n* = 14). Table 1 summarizes the demographic characteristics of the program users. Regarding the service providers, two recently joined the TBI program over the last year, four had been working in the program between 2 and 8 years, while the manager and the coordinators each had over 10 years of experience with the program.

**Table 1.** Demographic information of program users (*n* = 5).

Participant	Gender (M/W)	Age (years)	Mechanism of injury	Time since injury (months)
1	W	33	MVC	28
2	W	51	MVC	24
3	W	40	Fall	30
4	M	62	Fall	27
5	W	51	Fall	13

M, man; MVC, motor vehicle collision; W, woman.

Table 2 summarizes the eight overarching categories from the SWOT analysis: (1) *PA intervention*; (2) *health-related outcomes*; (3) *clinical expertise*; (4) *knowledge translation; (*5) *communication, (*6) *users’ engagement*; (7) *resources*; and (8) *accessibility*. Details and salient quotes supporting each category are presented narratively in Table 2.

**Table 2.** Overarching categories emerging from SWOT perceived by service providers and users of the TBI specialized program (*n* = 14).

Overarching category	Description of overarching category and categories of perceptions	SWOT	Service providers (*n* = 9)	Users (*n* = 5)
PA intervention	The PA intervention is accessible, individualized, and includes diverse activities such as home programming and group-based activity, but needs more structure, specificity, and clear goals to enhance service quality			
	PA intervention goals and exercise sessions are individualized and adaptable to an individual’s symptom status	S	✓	✓
	Group information sessions about resuming PA with mTBI and group-based adapted PA such as dance therapy and adapted collective sports offered in the rehabilitation center provide socialization opportunities and foster interactions between users	S	✓	✓
	Home programming is appreciated by users and allows them to stay active at home	S	✓	✓
	Access to clinicians responsible for PA intervention is easy and offers global management of mTBI	S	✓	
	PA session format and flexible scheduling are adequate	S		✓
	PA intervention is not structured and is lacking home follow-ups	W	✓	
	Clinical experts’ schedules are limited, negatively impacting users	W	✓	
	PA intervention is general and is not adapted to user’s capacity and needs	W		✓
	PA intervention goals and the exercise prescribed are complex, unclear, and not understood by users	W		✓
	PA intervention may be stopped before reaching rehabilitation goals	W		✓
	Stopping PA intervention can create bereavement feelings for users	W		✓
	There are delays for users to obtain interdisciplinary treatments	W		✓
	Adding new activities such as relaxation, yoga, and swimming could complement and improve the PA intervention	O	✓	✓
	Provide more opportunities for direct or indirect monitoring such as maintaining access to sports facilities during user transition periods upon arrival, during, and at the end of the program	O	✓	✓
	Offer more group and individual exercise sessions before, during, and after interdisciplinary interventions	O	✓	✓>
	Transfer users at the end of the PA intervention to community organizations specializing in adapted PA	O	✓	
	All users should have access to kinesiology and the PA intervention.	O		✓
Health-related outcomes	PA intervention improves users’ function and participation			
	PA intervention allows users to surpass themselves, to improve health-related outcomes such as self-confidence and mood, in addition to facilitating return to normal daily activities	S	✓	✓
	Users having participated in group intervention sessions created a peer-led support group which frequently met outside of the program	S		✓
Clinical expertise	Clinicians’ interpersonal and professional competence help deliver adapted PA intervention and humanist care			
	Clinicians’ expertise allows offering specific PA interventions adapted to users’ needs and capacities	S	✓	✓
	Clinicians’ interpersonal competences such as listening, being energetic, and empathetic contribute to providing humanist care and foster good relationships	S	✓	✓
	Staff turnover may create situations where clinicians responsible for the PA intervention have less experience to provide evidence-based treatment	W		✓
	Some clinicians are not motivating and lack empathy about persisting symptoms.	W		✓
	The beliefs, physical and intellectual limitations, occupations, and symptoms of users can interfere with the performance of the PA intervention	T	✓	✓
Knowledge translation	Knowledge translation about PA for an individual with a mTBI enhances services quality			
	Lack of time for professional development and program quality improvement efforts	W	✓	
	Developing processes to transfer evidence-based knowledge to clinicians would enrich their knowledge and improve PA intervention delivery	O	✓	
	Collaborating with researchers and clinicians specialized in oculovestibular therapy outside of the program could enhance PA intervention quality	O	✓	
	Sharing TBI specialized expertise in PA with physicians and other rehabilitation centers locally	O	✓	
	Lack of knowledge about health-related effects of mTBI and its management by external organizations (e.g., insurance companies, physicians) reduces referrals to program and access to PA intervention	T	✓	✓
Communication	Communication between clinicians and users ensures interdisciplinarity services and contributes to communicating a single vision about PA to users			
	Clinicians work in interdisciplinary teams allowing them to elaborate common treatment goals and communicate a single vision about PA	S	✓	✓
	Clinicians’ offices are strategically situated to facilitate interprofessional communication and PA intervention	S	✓	
	Clinicians have access to various tools fostering interprofessional communication	S	✓	
	Administrative agent offers critical support to clinicians for coordinating services	S	✓	
	Overlaps in professional roles of clinicians responsible of PA intervention generate disagreements	W	✓	
	Clinicians lack communication among themselves which provides users conflicting messages about PA	W		✓
Users’ engagement	Engaging program users in decision-making and responsibility sharing may improve PA participation.			
	Users could receive more information about possible risks of group-based adapted PA and consent to participate	O	✓	✓
	Creating a contract with users before admission to the program could consolidate their commitment to the PA intervention	O	✓	
	Mandatory participation in an information group when users are admitted to the program could ensure that all users receive the same information about PA before starting treatments	O	✓	
	Possibility of creating a peer-led support group that is not supervised by a clinician	O		✓
Resources	Adequate human, financial, and material resources ensure PA delivery			
	Infrastructures such as the pool, the exercise room, and the adapted break room allow clinicians to offer varied, adapted, and adequate PA interventions to users	S	✓	✓
	Exercise room is not adapted to deliver safe and efficient PA intervention	W	✓	✓
	Access to the swimming pool is limited, decreasing treatment options for clinicians	W	✓	✓
	Lack of personnel impairs PA intervention delivery to users	W	✓	✓
	Some exercise equipment needs, maintenance, or replacement, limiting PA interventions	W		✓
	User’s home equipment may not be adequate to perform at-home prescribed exercise	W		✓
	Job insecurity, lack of human resources, and staff turnover can undermine the expertise of the program and affect PA intervention delivery	T	✓	✓
	Budget restriction can affect the PA intervention delivery	T	✓	
	Gymnasium, some equipment, and spaces useful for PA intervention will be lost after the relocation	T	✓	
Accessibility	Users have access to a well-located rehabilitation center allowing community-based PA and real-life scenario interventions			
	Rehabilitation center’s geographic location increases accessibility, and it is well situated to provide community-based PA and real-life scenario interventions	S	✓	
	Upcoming relocation of the specialized program generates uncertainty about accessibility to PA intervention.	T	✓	✓

mTBI, mild traumatic brain injury; O, opportunity; PA, physical activity; S, strength; SWOT, strength, weakness, opportunity, and threat; T, threat; TBI, traumatic brain injury; W, weakness; ✓, Yes.

### PA intervention

The PA intervention at the time of the study was found to be accessible, individualized, and to include diverse activities such as home programming and group-based PA. The PA intervention format (i.e., individual and group-based interventions, home-programing) and the PA intervention’s flexible schedule were considered strengths by both service providers and program users. Service providers felt positive about the adequateness of the PA goals given to the users and the patient-oriented approach focusing on resolving individual symptoms. Moreover, group-based interventions and home programs were considered important components by service providers and program users. However, both groups identified several weaknesses such as needing more structure, specificity, and clearer PA goals to enhance service quality. For instance, one service provider thought that the PA intervention was less structured and “systematic” and more “random” in comparison to other interventions delivered within the specialized rehabilitation program. Similarly, some users felt the PA intervention was too general, lacked clarity with regard to targeted goals in PA and a clear exercise prescription. Interestingly, the endpoint of the intervention did not seem to be clear for users, with some users reporting that the PA intervention could be stopped before attaining rehabilitation goals. One user even reported having felt bereaved when stopping the PA intervention. Both service providers and program users recognized there were opportunities to add new exercise modalities, to provide more individual or group-based direct and indirect monitoring, and to improve transitioning of users into community-based organizations offering adapted PA. No threats were reported within this category.

### Health-related outcomes

The PA intervention was perceived to improve users’ function and foster their return to normal activities. This overall positive impact on health-related outcomes was perceived as a strength by both groups. Indeed, they reported the PA intervention provides multiple benefits for users, such as improving their energy, mood, self-confidence, and facilitating their return to normal activities. One user reported having improved self-confidence due to the PA intervention and the close supervision of the therapist: “The fact that, specifically, I didn’t have much trust in my balance, my therapist came with me outside to bicycle. This really helped me because it gave me back my confidence that I was able to do it [biking].” Users reported social benefits of the PA intervention group format in creating relationships and bonds outside of the program. Users reported creating a small peer-led support group meeting every other week outside of the program where they could share their experiences without the fear of being judged and where they did not need to justify themselves about their incapacities. No weakness, opportunity, or threat was reported.

### Clinical expertise

Clinical experts’ knowledge and interpersonal skills (e.g., listening, being energetic, being empathetic) were perceived to help promote the delivery of the PA intervention in a caring manner. Indeed, both program users and service providers thought the combination of these qualities fostered good relationships and promoted the delivery of specific PA-related interventions adapted to users’ needs. However, when these qualities were perceived to be less present, due to a lack of expertise or experience because of staff turnover, clinical expertise was reported as a weakness of the PA intervention. For instance, users expressed the lack of empathy about the persisting symptoms of fatigue, while another user expressed the inability of a clinician to motivate them. Interestingly, some characteristics of the users such as their beliefs, physical and intellectual limitations, occupations, or symptoms are considered threats to the PA intervention by both service providers and program users. For example, users may have concomitant injuries, post-traumatic stress disorders, or kinesiophobia needing particular attention: “Sometimes, it depends on their injury. If they have a musculoskeletal injury, it may limit [the intervention]. So, we have this to consider. Or, also, if they have post-traumatic stress disorder sometimes or kinesiophobia. This can alter [the intervention], in the sense that some are afraid of movement. . ..” The clinicians would then have to work around users’ characteristics to offer an adapted PA intervention which is considered less beneficial than if the intervention was not adapted. No opportunities were reported.

### Knowledge translation

Translating knowledge about PA for individuals with a mTBI was believed to have an impact on service quality and accessibility to the program. Indeed, service providers felt that their rehabilitation center had challenges translating evidence-based knowledge about PA for individuals with a mTBI *within* their rehabilitation program and difficulty promoting their clinical expertise *outside* the rehabilitation center. Moreover, they considered the lack of time for professional development or quality improvement efforts as a weakness that hindered PA intervention delivery. For instance, one service provider reported that “It is hard to free all clinicians’ schedule for one afternoon and think (together) about how to organize this component [PA intervention] of the program. It’s the users that are not being seen during this time.” Service providers felt they should develop strategies to translate evidence-based knowledge more efficiently to clinicians to enrich their clinical expertise and improve PA intervention delivery. Similarly, fostering collaboration with researchers within the institution and/or with specialized service providers outside of the program could enhance knowledge-sharing capacity and ultimately improve the quality of the PA intervention and accessibility to the program. Another perceived opportunity included sharing the program’s expertise about PA and mTBI with physicians outside of the program and other local rehabilitation centers to enhance program referral. There was also a shared perception about the lack of knowledge among external organizations (e.g., insurance companies, physicians) about the health-related effects of mTBI and the specialized management required to treat mTBI. This lack of knowledge was thought to be responsible for low numbers of program referrals and therefore reduced access to the PA intervention. No strengths were reported for this category.

### Communication

Service providers reported working in interdisciplinarity to elaborate common treatment goals and to communicate a common message about PA to users. Communication between service providers in this manner was generally perceived as a strength by service providers and some program users. Service providers also reported that their physical work environment (i.e., offices located near each other) and technological communication tools fostered good formal and informal communication. Despite the overall perception of good communication between the service providers, two main communication issues (weaknesses) were reported. First, the overlap of professional roles of service providers from different disciplines (e.g., physiotherapy and kinesiology) created tension between colleagues and disagreements. Second, contrary to the perception of the team’s capacity to elaborate and communicate a clear message about PA to their users, conflicting messages communicated to users were mentioned. One user reported being frustrated by a miscommunication about the termination of his PA intervention when he thought he was continuing the services: “I think it is terrible! And then they told me: ‘*Well, she planned to end the [PA] intervention with you*.’ Well. . . she hasn’t told me that, she even said that we would probably try to do some biking outside soon.” No opportunities or threats were reported.

### Users’ engagement

Service providers and program users reported wanting to improve user’s engagement in the PA intervention. Both groups saw several opportunities to improve responsibility sharing and shared decision-making as a means to improve users’ engagement. For example, service providers suggested that all users should be educated about PA and its potential benefits for individuals with a mTBI using a small group format to ensure everyone receives the same information before starting the PA intervention. Furthermore, one service provider suggested creating a detailed contract with each user to foster adherence to the PA intervention. Finally, both service providers and program users thought users should receive more information about the “risks” associated with being involved in group-based PA performed at the rehabilitation center (e.g., falls, body collisions, head impacts). One user suggested that written informed consent could be obtained from users to demonstrate their understanding and commitment to this PA intervention modality. No strength, weakness, or threat was reported.

### Resources

Service providers and program users reported that adequate human, financial, and material resources are crucial to support the PA intervention delivery. Both groups reported that the infrastructure, including the swimming pool, the exercise room and its equipment, and the adapted break room for relaxation, was a great strength of the program. It allows service providers to offer varied, adapted, and appropriate interventions. However, service providers and program users shared several weaknesses about available resources. To begin, some service providers and program users indicated that treatment options were limited because of restricted access to the swimming pool. One user reported feeling at risk of an injury using inadequate exercise equipment: “There is one stationary bike that I was using at the beginning that had an electrical resistance, then at one point, it stopped working. I was pedaling without any resistance. So, it puts you at risk for an injury.” In the same line of thought, users reported not having adequate equipment at home to perform prescribed exercises, which may hinder their adherence to the PA intervention. Service providers and program users shared the perception that staff turnover, reduced staff, and lack of job stability were threats to the PA intervention. A service provider felt the intervention was vulnerable to staff changes: “Each time we change personnel, each time we are losing expertise. So that is a threat for the PA intervention in particular.” Budget restrictions were also perceived as a threat by service providers who were directly involved in the PA intervention: “Yeah. . . If there is less budget, well it’s possible that they cut (days of work) in certain professions such as mine or kinesiology (. . .).” The upcoming relocation of the specialized program was perceived as a threat because of potential loss of access to adapted gymnasiums, equipment, and outdoor spaces all used to support the PA intervention. No opportunities were reported.

### Accessibility

Service providers and program users both highlighted the importance of having access to a well-located and easily accessible rehabilitation center. The geographic location of the rehabilitation center at the time of the study was considered a strength as it allows community-based PA and real-life scenario interventions. For example, one service provider reported the ease of delivering the PA intervention outside in parks around the rehabilitation center. However, the relocation of the TBI program to a different rehabilitation hospital in another part of the city was perceived as threatening the accessibility to the TBI program and consequently, to the PA intervention. Service providers were uncertain/worried about the ease of user access to the new location (e.g., less available parking, reduced public transportation, further from the current location’s catchment area) and the available environment for their PA intervention (e.g., swimming pool, parks). Users also reported being worried that the relocation would negatively impact the accessibility of program services. One user reported believing that the relocation might force some current or future users to be less adherent to treatments or to abandon them: “Well, there will be more and more people that will just abandon, or they won’t go to their treatments.” No weakness or opportunities were reported.

## Discussion

This is the first study to report on the perceptions of key stakeholders about an interdisciplinary outpatient PA intervention for adults with a mTBI delivered within a specialized TBI rehabilitation program. These results are important, given the increasing evidence about potential benefits of PA for persons with mTBI,^14^ the growing interest within the province of Québec in creating evidence-based PA interventions aligning with user needs,^18^ and for promoting evaluations of health services for individuals with a TBI.^34^ Many strengths and weaknesses, several opportunities, and few threats of the outpatient PA intervention for individuals with a mTBI were identified. These perceptions provide new grounds to promote change, development, and quality improvement of PA interventions for outpatient adults with a mTBI both locally and globally.

There were convergent opinions about the general appreciation of some intervention characteristics (e.g., accessible, individualized, group-based), divergent opinions were also reported about aspects of the PA intervention (e.g., adequateness of the intervention, clarity of goals, exercise prescription, home program feasibility, miscommunication between clinical experts). Service providers and program users both shared their perspective about factors external and internal to the rehabilitation program (e.g., accessibility, resources, knowledge translation), and factors related to the individuals and their relationships (e.g., user engagement, clinical expertise, communication) as structured by the Consolidated Framework for Implementation Research (CFIR).^35^ This finding contributes to the field of implementation sciences and quality improvement, suggesting that a PA intervention embedded within an interdisciplinary outpatient program represents more than the exercise prescription parameters themselves.

Interestingly, important characteristics about the current PA intervention were not clearly understood by service providers and program users. Specifically, it appears that the therapeutic goals of the PA intervention and the exercise parameters are individualized and adapted to user-specific needs, which may explain the perceived “random” and “variable” nature of the goals and exercise prescription. Moreover, clinical managers and researchers identified at the outset of this project that the PA intervention was not formally documented, and exercise prescription parameters were not defined. This most probably contributed to this perception, since without clear goals and a replicable intervention, users may not receive an adequate PA intervention. To help clinicians improve PA delivery and their exercise prescription, future research should aim to uncover the optimal exercise parameters (e.g., length of intervention, frequency, intensity, duration, type of exercise, and progression).

Despite lack of clarity about what was being delivered to users, the PA intervention is perceived by stakeholders to improve some health-related outcomes. These perceived benefits align with literature supporting the effectiveness of PA on mood and return to normal activities and add to an emerging literature supporting improvement of self-esteem following mild TBI.^11,20,21,36–39^ Remarkably, neither service providers nor users reported the PA intervention as a means to reduce persisting symptoms of a mTBI, although recent systematic reviews and meta-analyses indicate that PA, and more precisely cardiovascular exercise, is effective in reducing persisting symptoms of a mTBI and hastens return to normal activity.^10^ There are multiple ways of interpreting these results. It is plausible that service providers have different beliefs, are unaware, or are not comfortable with this new evidence-based approach for this population, or they may have distinct rehabilitation goals other than reducing persisting symptoms. Knowledge translation challenges identified by this study could explain this. Future research of our research team could investigate if enhancing the clarity of PA intervention goals and approaches is linked to overall improvement in perceptions about the PA interventions by both services providers and program users.

An unexpected outcome of the PA intervention identified in this study was the creation of a peer-led support group by users participating in group-based PA activities. This may demonstrate the positive impacts of group dynamics, but more importantly, further highlight the social needs of adults with persistent symptoms of mTBI to socialize and share experiences among peers.^40^ Literature about the potential benefits of peer-led support groups for individuals with mTBI is scant; however, there is growing evidence about how peer-led programs may provide multiple social benefits and improve health-related outcomes among individuals with moderate and severe TBI, stroke, and spinal cord injury.^41–43^ Service providers may want to explore formally including a peer-support aspect in their PA intervention.

An important upcoming challenge reported by service providers is maintaining the access of the PA intervention after their relocation to a rehabilitation hospital beyond the current catchment area. Users included in this study feared that the move could lead users to abandon their interventions. Indeed, the geographic location within reasonable proximity of a health service is a well-known barrier to healthcare access and may lead to a diverse set of negative consequences (e.g., physical, psychological, social, and economic consequences) if individuals have reduced access.^44^ However, the location of a health service is not the only dimension of access to health services. In addition to accessibility, the availability, acceptability, affordability, the adequacy, and awareness are all dimensions of access.^45^ The service providers may explore solutions to maintain or enhance the access of their PA intervention in the upcoming quality improvement efforts. For example, as users reported appreciating the home-based PA component of the PA intervention, service providers could potentially improve accessibility, adequacy, and acceptability by offering enhanced home programs or adjunct telerehabilitation services to increase or maintain adherence, particularly during a pandemic.

## SWOT analysis

This study supports the use of SWOT analysis as a promising tool to uncover the perceptions of key stakeholders for quality improvement. During a relatively short interview with each stakeholder, we were able to understand the perceptions about the PA intervention. The results of the study also support involving program users in SWOT analysis; most studies reporting SWOT results in health care only include the perspective of providers^26,27^ or patient associations (e.g., user *needs*), not specifically program users (e.g., user *experiences*).^46^ Partnering with stakeholders such as service providers and program users is a key approach to improve meaningfulness and understandability of the results.^47,48^

### Recommendations

This study was conducted to better understand an existing PA intervention to guide future quality improvement efforts. Indeed, implementation of change often fails because of barriers at multiple levels within a health organization (e.g., patient level, provider team level, organizational, or policy level).^35^ Understanding of the inner workings and outer setting of a health organization helps to identify potential barriers, can guide selection of effective interventions, and ultimately improve strategical planning of quality improvements. This study helped identify areas of the current PA intervention to maintain and others that could be improved upon. It provides a strong rational for the use of implementation framework (e.g., CFIR) to structure change and quality improvement efforts for outpatient programs seeking to enhance PA. Involving both service providers and program users with a mTBI during the upcoming quality improvement efforts is recommended to improve their knowledge and skills and to increase the relevance of the changes and foster their implementation.^40,41,49^

Three main recommendations stem from the study. First, to enhance and evaluate their current services, the clinical program should better describe the underlying intervention theory of the existing PA intervention, its processes, and its goals.^50^ Indeed, this is pertinent for all rehabilitation programs wanting to create a PA intervention within their program. To help describe the PA intervention and plan quality improvement efforts, service providers should build a logic model; a visual representation of the relationships between resources, process, and outcomes of a health intervention.^50^ Mobilizing enough time and resources to generate a logic model of the PA intervention may be challenging for a clinical program that reports time constraints for knowledge translation and quality improvement efforts. Moreover, service providers should use an intervention replication checklist such as the Template for Intervention Description and Replication^51^ or the Consensus on Exercise Reporting Template^52^ to help describe in detail the specific parameters of their PA intervention. Besides ensuring a standardized and replicable intervention for all users, service providers would then be able to compare their PA intervention to most of the recent evidence and thus make appropriate choices about evidenced-based approaches to implement.

Second, to improve the knowledge translation capacity of the specialized program, identifying solutions to overcome this barrier, such as embedding quality improvement activities in normal clinical workflow^19^ or obtaining funding to support partial release of clinical work of key clinical experts, could be envisaged. Moreover, involving professionals with research and communication skills such as knowledge brokers and/or consolidating research and quality improvement activities with researchers could help improve service quality and promote evidence-based practice.^19,53^ Considering existing challenges about implementation of evidence-based practice in health care,^35^ we believe this is relevant to similar programs seeking to improve their clinical services.

Third, although we were originally pleased by how service providers openly shared their perspectives about the current PA intervention, we believe it is also important to assess the program’s organizational readiness for a change. Indeed, identifying areas of improvement with a SWOT analysis may orient improvement efforts, but a willingness to participate in an interview does not mean that stakeholders are ready to change their practices. A standardized questionnaire such as the *Organizational Readiness for Knowledge Translation Questionnaire* could be grafted for a SWOT analysis conducted elsewhere to better inform improvement efforts.^54^

### Study limitations

The results of this study, based on the perspectives about a local program and intervention, may be somewhat transferable to other TBI rehabilitation programs within the province of Québec but limited outside of the province where health ministry and automobile insurance board funding may differ. However, since to our knowledge, no interventions for persons with persisting symptoms have been described in detail in the literature, the results could assist other outpatient rehabilitation programs to consider using frameworks (e.g., CERT, SWOT analysis, CFIR, logic modeling) to create new PA interventions or to enhance existing PA interventions for adults with persisting symptoms of a mTBI. It is plausible that involving the service providers in this study might have influenced how they conceptualized the PA intervention while they shared their perceptions. Indeed, it would have been optimal to have a standardized intervention about which the stakeholders could have commented. However, there was no documented information, and no audit could be performed to see if the intervention was provided as per protocol. As clinicians often formally or less formally rethink, adjust, adapt, and modify their intervention provided to their users, we are aware that perceptions of stakeholders about the overall PA intervention provided in the program may not represent what was performed, but that their perceptions are still valuable. Although saturation may not have been attained because of the limited number of study participants, the combination of the recruitment challenges, the specificity of the sample, and the quality of the dialogue with the adult users with a mTBI justified the small sample without reducing the “power” of the information gathered.^31^ It is plausible that the minor adjustments to the interview guides and the probing depth provided by the senior scientist (BS) influenced how participants responded to the questions. We believe the adjustments may have improved the outcomes of the interviews (i.e., it enhanced the overall quality of the interviews and therefore, the depth and breadth of the answers provided by the participants). Also, as in any similar study, a social desirability bias is possible; both users and service providers may have presented a more favorable picture of the PA intervention. However, the presence of conflicting and somewhat negative perceptions of the PA intervention indicates that at least some of the participants shared their critical opinions about the PA intervention, thus supporting limited social disability bias.

## Conclusions

This study successfully used a SWOT analysis to identify strengths, weaknesses, opportunities, and threats related to the quality of a PA intervention within a specialized TBI rehabilitation program. It provides key insights about components of the PA intervention to maintain or to build upon during upcoming quality improvement efforts. The results can help inform PA intervention creation, enhancement, or adoption by other specialized outpatient programs seeking to implement new effective PA approaches for individuals with a mTBI that consider program user needs.

## Supplemental Material

sj-docx-1-smo-10.1177_20503121231166638 – Supplemental material for SWOT analysis of a physical activity intervention delivered to outpatient adults with a mild traumatic brain injury Click here for additional data file. Supplemental material, sj-docx-1-smo-10.1177_20503121231166638 for SWOT analysis of a physical activity intervention delivered to outpatient adults with a mild traumatic brain injury by Christophe Alarie, Isabelle Gagnon, Lily Trang Thao Huynh, Karine Doucet, Adèle Pichette-Auray, Cassandre Hinse-Joly and Bonnie Swaine in SAGE Open Medicine

sj-docx-2-smo-10.1177_20503121231166638 – Supplemental material for SWOT analysis of a physical activity intervention delivered to outpatient adults with a mild traumatic brain injury Click here for additional data file. Supplemental material, sj-docx-2-smo-10.1177_20503121231166638 for SWOT analysis of a physical activity intervention delivered to outpatient adults with a mild traumatic brain injury by Christophe Alarie, Isabelle Gagnon, Lily Trang Thao Huynh, Karine Doucet, Adèle Pichette-Auray, Cassandre Hinse-Joly and Bonnie Swaine in SAGE Open Medicine

sj-docx-3-smo-10.1177_20503121231166638 – Supplemental material for SWOT analysis of a physical activity intervention delivered to outpatient adults with a mild traumatic brain injury Click here for additional data file. Supplemental material, sj-docx-3-smo-10.1177_20503121231166638 for SWOT analysis of a physical activity intervention delivered to outpatient adults with a mild traumatic brain injury by Christophe Alarie, Isabelle Gagnon, Lily Trang Thao Huynh, Karine Doucet, Adèle Pichette-Auray, Cassandre Hinse-Joly and Bonnie Swaine in SAGE Open Medicine

sj-docx-4-smo-10.1177_20503121231166638 – Supplemental material for SWOT analysis of a physical activity intervention delivered to outpatient adults with a mild traumatic brain injury Click here for additional data file. Supplemental material, sj-docx-4-smo-10.1177_20503121231166638 for SWOT analysis of a physical activity intervention delivered to outpatient adults with a mild traumatic brain injury by Christophe Alarie, Isabelle Gagnon, Lily Trang Thao Huynh, Karine Doucet, Adèle Pichette-Auray, Cassandre Hinse-Joly and Bonnie Swaine in SAGE Open Medicine
