# Supplementary material for: SWOT analysis of a physical activity intervention delivered to outpatient adults with a mild traumatic brain injury
Source: SAGE Open Med. 2023 Apr 17;11:20503121231166638. doi: 10.1177/20503121231166638 (PMC10123884; doi:10.1177/20503121231166638)
Supplement: sj-docx-2-smo-10.1177_20503121231166638 – Supplemental material for SWOT analysis of a physical activity intervention delivered to outpatient adults with a mild traumatic brain injury [file sj-docx-2-smo-10.1177_20503121231166638.docx]

**Supplementary File I**

**SWOT analysis of a physical activity intervention delivered to outpatient adults with a mild traumatic brain injury (mTBI)**

**Interview Guide – Clinicians**

**1. Demographics**

a. Can you describe your position and role in the program?

b. How long have you been in this profession?

c. How long have you been working with mTBI clients?

**2. Strengths of physical activity intervention**

a. In your opinion, what are the strengths of the current physical activity intervention provided to the program’s mTBI clientel?

i. Probe with: Strengths regarding the type of training, frequency of supervised sessions with clinicians, motivation, schedule, location, supervision style, group intervention, environment, activity choices, etc.

b. What do you think this approach can do for the program users?

i. Probe with: Symptoms, fatigue, pain, participation, etc.

**3. Weaknesses of the physical activity intervention**

a. In your opinion, what are the weaknesses of the current physical activity intervention?

i. Probe with: Strengths regarding the type of training, frequency of supervised sessions with clinicians, motivation, schedule, location, supervision style, group intervention, environment, activity choices, etc.

b. Did you perceive any negative effects for users?

i. Probe with: Fatigue, pain, dropping out of activities, etc.

**4. Threats of the physical activity intervention**

a. What do you think might interfere with consumers' participation in the current physical activity intervention?

i. Probe with: Lateness/absenteeism, non-compliance, infrastructure, equipment, scheduling, group training, etc.

**5.Opportunities for the current physical activity intervention**

a. What could improve or enhance the current physical activity intervention?

b. How do you think consumer participation could be optimized?

i. Probe with: Type of activity, schedule, environment, supervision, frequency?

c. Based on what you mentioned earlier (difficulty X mentioned above), do you have any ideas for potential solutions?

**6.Conclusion**

a. Is there anything else you would like to add to our discussion?

**Field notes:**
