# Supplementary material for: SWOT analysis of a physical activity intervention delivered to outpatient adults with a mild traumatic brain injury
Source: SAGE Open Med. 2023 Apr 17;11:20503121231166638. doi: 10.1177/20503121231166638 (PMC10123884; doi:10.1177/20503121231166638)
Supplement: sj-docx-3-smo-10.1177_20503121231166638 – Supplemental material for SWOT analysis of a physical activity intervention delivered to outpatient adults with a mild traumatic brain injury [file sj-docx-3-smo-10.1177_20503121231166638.docx]

**Highlights**

**1. What do we already know about this topic?**

Physical activity interventions can improve function and promote participation of adults with a mild traumatic brain injury but not all programs provide these interventions.

**2. How does your research contribute to the field?**

Identifying strengths, weaknesses, opportunities, and threats related to the quality of a physical activity intervention of a specialized TBI rehabilitation program, help target intervention components for local and widespread service delivery improvement.

**3. What are your research’s implications towards theory, practice, or policy?**

SWOT analysis is helpful to inform rehabilitation and physical activity intervention development but should be complemented with theoretically driven models to better direct quality improvement efforts.
