# Supplementary material for: SWOT analysis of a physical activity intervention delivered to outpatient adults with a mild traumatic brain injury
Source: SAGE Open Med. 2023 Apr 17;11:20503121231166638. doi: 10.1177/20503121231166638 (PMC10123884; doi:10.1177/20503121231166638)
Supplement: sj-docx-4-smo-10.1177_20503121231166638 – Supplemental material for SWOT analysis of a physical activity intervention delivered to outpatient adults with a mild traumatic brain injury [file sj-docx-4-smo-10.1177_20503121231166638.docx]

**Supplementary File I**

**SWOT analysis of a physical activity intervention delivered to outpatient adults with a mild traumatic brain injury (mTBI)**

**Interview Guide – Program users**

**1. Demographics**

a. What is your age?

b. What is your current occupation and what was it at time of your injury?

c. What was the mechanism of your injury?

d. What was the date of your injury?

e. Was this your first head/brain injury?

**2. Physical activity intervention**

a. When were you been admitted to the TBI program?

b. Can you describe the physical activity intervention you received?

i. Probe with: which professional, duration, intensity, frequency, mode of physical activity, nature of counselling, difficulty, etc.,

**2. Strengths of physical activity intervention**

a. In your opinion, what are the strengths of the physical activity intervention you received? In other words, what did you like about the intervention?

i. Probe with: Strengths regarding the type of training, frequency of supervised sessions with clinicians, motivation, schedule, location, supervision style, group intervention, environment, activity choices, etc.

b. What, if any, were the benefits for you of the physical activity intervention?

i. Probe with: Symptoms, fatigue, pain, participation, etc.

**3. Weaknesses of the physical activity intervention**

a. In your opinion, what are the weaknesses of the physical activity intervention you received? In other words, what did you like the least about the intervention?

i. Probe with: Weaknesses regarding the type of training, frequency of supervised sessions with clinicians, motivation, schedule, location, supervision style, group intervention, environment, activity choices, etc.

b. Have you perceived any negative effects or experienced adverse events from the physical activity intervention?

i. Probe with: Fatigue, pain, dropping out of activities, etc.

**4. Threats of the physical activity intervention**

a. Were you always able to do the physical activities that were planned or suggested?

b. What do you think might interfere with the physical activity intervention you received?

i. Probe with: Lateness/absenteeism, non-compliance, infrastructure, equipment, scheduling, group training, etc.

**5.Opportunities for the current physical activity intervention**

a. What could improve or enhance the current physical activity intervention?

b. How do you think your participation in the physical activity intervention could be optimized?

i. Probe with: Type of activity, schedule, environment, supervision, frequency?

c. Based on what you mentioned earlier (difficulty X mentioned above), do you have any ideas for potential solutions?

**6.Conclusion**

a. Is there anything else you would like to add to our discussion?

**Field notes:**
